# Is there a potential role for CMR in deciding appropriateness for ACID placement?

**DOI:** 10.1186/1532-429X-17-S1-P229

**Published:** 2015-02-03

**Authors:** Hayah Kassis, Brandon M Mikolich, Robert W Biederman, Ronald J Mikolich

**Affiliations:** Cardiology, Allegheny General Hospital, Pittsburgh, OH USA; Cardiology, Sharon Regional, Sharon, PA USA

## Background

It is a class I indication to place AICDs in patients with ischemic or nonischemic cardiomyopathy with a left ventricular ejection fraction (LVEF) ≤ 35%. Most commonly, the need is determined by LVEF measurements on 2-D echo. This practice was utilized prior to cardiac MRI (CMR) establishment as the LVEF "gold standard" modality. Transthoracic echocardiography is still widely employed because of its wide availability and perceived cost savings. This study was designed to compare LVEF by both 2-D and CMR prior to AICD, along with analysis of comparative Medicare reimbursement.

## Methods

Patients in a multi-institutional cardiac imaging database who underwent AICD implantation according to class I guidelines were queried for pre-AICD 2-D echo and CMR exams, constituted the study population. LVEF by 2-D and CMR were assessed for concordance for LVEF < 35%. The discordant patients were categorized as higher or lower CMR LVEF, relative to 2-D LVEF. Using 2012 Medicare reimbursement data for Western Pennsylvania, imaging reimbursement prior to AICD implantation per 100 patients was calculated.

## Results

131 patients met entry criteria for this study. Seven of 131 patients (5.3%) had LVEF > 35% on CMR, but ≤ 35% on 2-D. Eleven of 131 patients (8.4%) had LVEF ≤ 35% on CMR, but > 35% on TTE. Overall, 18 of 131 patients (13.7%) showed discordance between CMR and 2D and may have had an incorrect decision for AICD based on 2-D echo alone. The reimbursement of a Structure/Function CMR exam (codes 75557 and 75565) was $30,653 per 100 patients, while 2-D/Doppler exam (codes 93325 and 93306) was $35,578 per 100 patients.

## Conclusions

Of patients undergoing AICD implantation for primary prevention, 5.3% had an LVEF > 35% on CMR, and may not have required an AICD. Conversely, 8.4% of patients would not have received an AICD based on 2-D results. For patients with cardiomyopathy undergoing assessment for the need of AICD for primary prevention, CMR is likely more accurate and is a cost saving imaging strategy for evaluation of AICD implant candidates with current reimbursement rates in the United States.

